# Give Us Back Our 11 Days

**Published:** 1992-08

**Authors:** Hamish Thomson

**Affiliations:** Consultant Surgeon The Gloucester Clinic


### Give us Back our 11 Days

**Hamish Thomson, M.S., F.R.C.S.**
Consultant Surgeon
The Gloucester Clinic

It has long been a matter of idle speculation with me to wonder what kind of lives the sort of people who are terribly good at something or other led before the something or other they turned out to be terribly good at were invented. To make myself clearer, how must chaps like Chopin or Art Tatum, say, have felt before the piano came on the scene, or Lord Lichfield before photography? What the eye doesn't see the heart may not grieve after, but surely they must have experienced an inexpressible yearning for they knew not what. Did their parents fret over the listless unrequited look in their eyes as they plodded desultorily about their daily tasks? It wouldn't be a bit surprising.

These musings were prompted by a little problem we have just had with our computer, because in that article you have embodied the very essence of the phenomenon. For thousands and thousands of years, since mankind first struggled from the primaeval swamp, computer-friendly people must have been born to generation after generation for whom there was no computer to befriend. There were their genes crying out for a keyboard, a mouse and a VDU, but like the immaculately groomed poodle on the day they cancelled Crufts, all dressed up with nowhere to go. As they hunted and gathered were they puzzled by an aching but intangible dissatisfaction? We'll never know. It was to be millenia before they found their niche in life. The abacus may have proved a welcome distraction for a bit but it can't have enchanted for long. It certainly wouldn't scratch the surface for the real computer buff as we know him today.

But here at last computers are to keep their tryst in the world's evolving story, and we were jolly glad to have someone around of that ilk who knew how ours worked when things went wrong.

As I said above we have recently had a bit of trouble. In a word, according to the pundits, it went 'down'. I am not familar with the jargon, being the computer equivalent of tone deaf (to continue the earlier musical metaphor), but so far as I understand 'down' means a sort of concussion. Like concussion there is usually no permanent damage to the little grey cells and apparently the phenomenon is common so we weren't to worry.

However, as the patient revived under the healing influence of our expert's attention and started talking to us again, realisation gradually dawned that the damage was deeper than at first suspected. Hardly had we wiped the brow in relief at our little slave's apparent recovery when we discovered that the liquid crystals (now there's a contradiction in terms to an ignoramus like me), twinkle though they never so greenly, were putting a brave face on a hidden loss. For during its few hours of incapacity - an apt epithet if computers had capacitors, which they probably don't - our machine had sustained an irrecoverable amnesia, shedding 11 days worth (well, roughly, but I couldn't resist the historical allusion) of labouriously garnered facts.

You would hardly believe it but its innards work like this: every time the secretary wants to add the last week's work to - pardon me, update - the floppy disc, the process involves transference of the disc's entire record to the computer first, so temporarily wiping the disc clean. There the new data is assimilated before the whole lot is passed back - sorry, downloaded - to the disc again. This happens every time, week in, week out, until the disc is full. With so much repeated erasure and re-charging, its no wonder the disc gets floppy.

So you can see how disaster could strike. Just as the magnetics had yielded their precious cargo to the electric circuitry for updating, something fused, incandesced, coalesced, or whatever, and part of the load ended up as a disorganised diaspora of evicted electrons. The old f.d. then got the cargo back, duly augmented at one end but missing a chunk in the middle.

The thing was, why? Could a jealous colleague have hacked into my data-base to extract the enviable results therein for purposes of plagiarisation? Was I, alternatively, part of a hapless pool of targeted milch-cows unknowingly providing statistics for a super-national surgical audit show-down? Surely Brendan Devlin wouldn't go that far.

No. It seemed more likely that we had got a bug. Not a computer "virus" of the sort we read, sewn into the processor by a boffin-brained prankster, but something altogether more mundane. My theory is that some denizen of the insect world had simply crept through a crevice or even walked boldy up the disc port lured no doubt by the warmth and seclusion afforded within. Winter was approaching so perhaps it planned to hibernate; if so its cosy hopes were cruelly shattered. You can imagine it snuggling in there, eyelids drooping preparatory to throwing up a few solid Z's, leg one on one bit of the micro circuit, leg two (both probably just licked clean and still moist from the creature's habitually fastidious pre-nap ablutions thereby ironically ensuring a sound electrical contact, so cruelly does divine retribution miss its target) on another and so on, just as the nature-loving, muesli-eating, wouldn't-hurt-a fly secretary threw the switch. If bigger, a centipede say, rather than the afore-mentioned fly, it might even have straddled two adjoining chips, a Collosus of Rhodes in miniature. Whatever size and however disposed, the ingredients of the cocktail were in place for the fateful moment when our weekly information transfer would make the connection, and so with a muffled "pop" simultaneously achieve both its own apotheosis and obliteration of the information which formed first its bed and then its sepulchre.

It probably led a dull and unpleasant life and I musn't begrudge it the glorious effulgent spark which announced its end. It exacted a heavy toll though, in going, taking a lot with it when it went. I've been bitten by lots of bugs. We all have. However this sort of bite is different. When a bug can remove a byte you've got to take prophylactic measures. From now on its a separate printed copy for me every week before we feed it to the total. As a chap I knew who used to live in Bitterne and didn't like it used to say, once bitten twice shy.

